# Review on *Trypanosoma cruzi*: Host Cell Interaction

**DOI:** 10.1155/2010/295394

**Published:** 2010-07-29

**Authors:** Wanderley de Souza, Tecia Maria Ulisses de Carvalho, Emile Santos Barrias

**Affiliations:** ^1^Laboratório de Ultraestrutura Celular Hertha Meyer, CCS, Instituto de Biofísica Carlos Chagas Filho, Universidade Federal do Rio de Janeiro, Bloco G, Ilha do Fundão, RJ 21941-902, Brazil; ^2^Diretoria de Programas, Instituto Nacional de Metrologia, Normalização e Qualidade Industrial—Inmetro, Av. Nossa Senhora das Graças, 50 Xerém-Duque de Caxias, RJ 25250-020, Brazil; ^3^Instituto Nacional de Ciência e Tecnologia em Biologia Estrutural e Bioimagens, Universidade Federal do Rio de Janeiro, Ilha do Fundão, RJ 21941-902, Brazil

## Abstract

*Trypanosoma cruzi*, the causative agent of Chagas' disease, which affects a large number of individuals in Central and South America, is transmitted to vertebrate hosts by blood-sucking insects. This protozoan is an obligate intracellular parasite. The infective forms of the parasite are metacyclic and bloodstream trypomastigote and amastigote. Metacyclic trypomastigotes are released with the feces of the insect while amastigotes and bloodstream trypomastigotes are released from the infected host cells of the vertebrate host after a complex intracellular life cycle. The recognition between parasite and mammalian host cell involves numerous molecules present in both cell types. Here, we present a brief review of the interaction between *Trypanosoma cruzi* and its host cells, mainly emphasizing the mechanisms and molecules that participate in the *T. cruzi* invasion process of the mammalian cells.

## 1. Introduction to *T. cruzi* and Its Life Cycle

Protozoa of the Trypanosomatidae family are agents of parasitic diseases that have a high incidence and a negative economic impact in developing countries. In the case of leishmaniasis, caused by several species of *Leishmania*, about sixteen million people are infected in Africa, Asia, parts of Europe, and Latin America. Sleeping sickness, caused by the *Trypanosoma brucei* group, affects about three million people in Africa. In the case of Chagas' disease, caused by *Trypanosoma cruzi*, sixteen to eighteen million individuals are infected and more than 80 million are at risk of infection (http://www.who.org/). Some trypanosome species are also important in veterinary medicine, since they have been seriously affecting animals of economic interest such as horses and cattle. Diseases caused by plant trypanosomatids are increasing in importance owing to the serious problems they have caused in coconut and oil palm plantations in South America.

**Figure 1:**
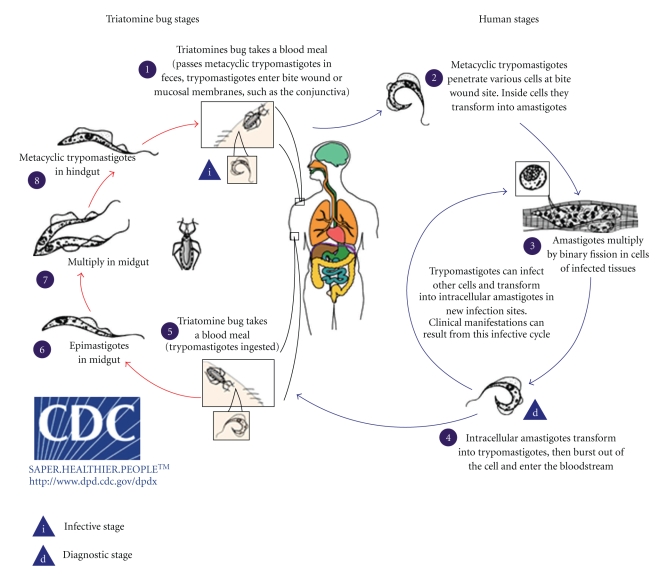
Life cycle of *T. cruzi* showing the various forms of the protozoan in the invertebrate (triatomines) and vertebrate (mammals) hosts. Figure reproduced from the Center of Control Diseases homepage.

 One specific feature of the trypanosomatids is that they change their general shape during their life cycle. In those species that switch from vertebrate to invertebrate hosts, this and other changes may be dramatic, involving the appearance of developmental stages which do not divide and stages which are highly infective through a process generally described as protozoan differentiation or transformation [1, 2]. Among the trypanosomatids, *T. cruzi* presents one of the most complex life cycles involving several developmental stages found in the vertebrate and the invertebrate hosts as well as in the bloodstream and within vertebrate host cells. Figure 1 shows a general view of the life cycle of this protozoan. Let us consider that the cycle starts with insects from the Reduviidae family sucking the blood of vertebrates infected with the trypomastigote forms which circulate in the bloodstream (known as bloodstream trypomastigotes). Once ingested, most of the trypomastigotes are lysed in the insect's stomach [3]. The surviving trypomastigotes transform, in a few days later, either into spherical stage (known as spheromastigotes) or into epimastigote stage. Epimastigotes migrate to the intestine where they divide intensely and attach to the perimicrovillar membranes which are secreted by intestinal cells of posterior midgut [4, 5]. This adhesion step seems to be important to trigger the process of transformation of the noninfective epimastigotes into highly infective trypomastigotes (known as metacyclic trypomastigotes). The adhesion process of epimastigotes to the perimicrovillar membranes involves the participation of surface-exposed glycoconjugates. Several proteins found in the perimicrovillar membranes seem to be involved in this process [4]. Also, surface glycoinositolphospholipids (GIPLs) of the parasite have been shown to be involved in the attachment process [5]. Several saccharides are able to inhibit parasite attachment.

At the most posterior regions of the intestine and at the rectum, many epimastigotes detach from the intestinal surface and transform into metacyclic trypomastigote forms which are then released together with feces and urine [6]. These stages are also designated as metacyclic trypomastigotes which are highly infective for several mammalian species, including human. Usually the infection of mammalian takes place through direct inoculation of these forms through the ocular mucosa or the lesioned skin during insect blood meal. Other important transmission mechanisms are by blood transfusion, transplacentary, and organ transplant. Nowadays these mechanisms are much less frequent due to vector control programs and careful analysis of blood donors. However, it has been shown quite recently that these stages are also infective through the oral route [7]. Once in the vertebrate host, the metacyclic trypomastigotes invade the cells at the inoculation site (e.g., fibroblasts, macrophages, and epithelial cells) through recognizing between parasite and vertebrate host cells in a process that involves a great variety of molecules present in both cell starting the intracellular life cycle of *Trypanosoma cruzi*. This cycle involves several steps like the formation of an endocytic vacuole known as the parasitophorous vacuole, a differentiation of the long and thin trypomastigote forms in rounded with a short flagellum, characteristic of the amastigote forms (also known as intracellular spheromastigotes) and lysis of the parasitophorous vacuole membrane by enzymes secreted by the parasite [8] so that the amastigote forms enter in direct contact with host cell organelles. Here, we will review all basic steps involved in the process of interaction of *T. cruzi* with cells both from the vertebrate and invertebrate hosts. We will focus mainly on the basic biological processes which take place in any kind of cell.

## 2. Adhesion of *T. cruzi* to Vertebrate Cells

The first steps of the *Trypanosoma cruzi*—host cell interaction process can be divided into three stages: adhesion and recognition, signaling, and invasion. In studies of *T. cruzi*—host cell interaction, we should consider: (i) the *T. cruzi* strain used in the studies [10], (ii) which developmental stage is used [10], (iii) whether the trypomastigote form used is slender or stout, and (iv) which host cell is used [11]. Therefore, it is possible to anticipate that the mechanisms involved on recognition, signaling, and invasion (or phagocytosis) are complex.

**Figure 2:**
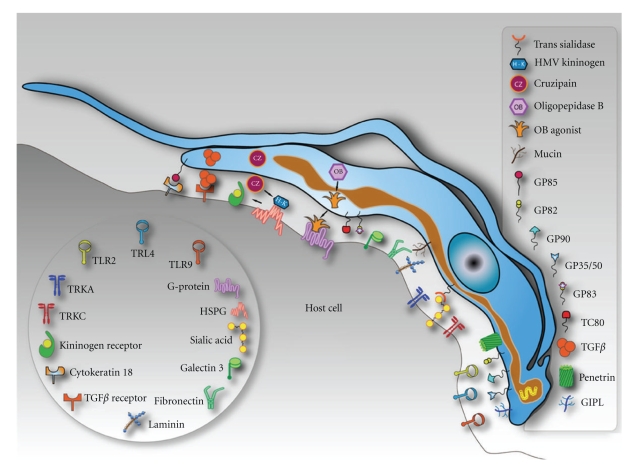
Schematic model summarizing the molecules involved on parasite-host cell interaction process and exposed on the surface of a hypothetical host cell and in trypomastigotes of *Trypanosoma cruzi. *

The adhesion step involves the recognition of molecules present on the surface of both parasite and host cells (Figure 2). We cannot exclude the possibility that molecules secreted by the parasite may also play some role in this process, as clearly shown in members of the Apicomplexa phylum. Adhesion and internalization are clearly different processes which can be separated. For example, when cells are allowed to interact at 4°C, only adhesion takes place [12]. Treatment with actin polymerization inhibitors, such as cytochalasins, also shows a clear picture of the adhesion step. Adhesion is a process that depends on receptors restricted to membrane domains. The adherence of the parasite to a host cell does not mean that invasion will take place. 

The mechanisms by which *T. cruzi* infective forms gain access to the intracellular milieu are gradually being disclosed. The involvement of parasite ligands and/or host cell receptors has been extensively studied [13–15]. In the next topics, we will review some *T. cruzi* and host cell molecules involved in the recognition of both cells.

## 3. Recognition Process in the Vertebrate Cell

### 3.1. Parasite Molecules

Different strains of *T. cruzi *as well as different forms of the parasite (tissue culture-derived trypomastigotes, metacyclic trypomastigotes and amastigotes), express different molecules on their surface. These surface molecules interact with host components to invade mammalian cells. First, we will comment on the trypomastigote surface molecules and then those present on amastigote.

In 1983, Nogueira [19] described, using macrophages as the host cell, the antiphagocytic effect of a gp90 molecule that is present in the mammalian stages of *T. cruzi. *This molecule seemed to have glycosidase activity and its antiphagocytic activity was mediated by the removal of sugar residues necessary for parasite internalization. The ability of metacyclic trypomastigote gp90 to downregulate host cell invasion has been associated to the lack of Ca^2+^ signal-inducing activity by this molecule. Intracellular Ca^2+^ mobilization, both in the target cell and the parasite, was required for *T. cruzi* internalization [13, 20]. Binding of gp90 to mammalian cells does not trigger these Ca^2+^ responses [21]. The gp90 molecule is easily digested by gastric juice (pepsin), thus explaining the observation that when isolates rich in gp90 were accidentally ingested the parasite entry in host cells and the infection was maintained [22]. *Trypanosoma cruzi* molecules known as mucins are also involved in host cell invasion. Mucins are the major *T. cruzi* surface glycoproteins and their sugar residues interact with mammalian cells [23, 24]. Many mucins have been implicated in host cell infections [24–26]. Schenkman and colleagues [27] reported that mucins can also act as ligands.

In 1986, an 85 KDa glycoprotein was identified as a ligand of fibronectin in different cell types like monocytes, neutrophils and fibroblasts [30, 31]. This glycoprotein is known as Tc85. The Tc85 molecule is abundant in trypomastigotes and is characterized as part of the gp85/trans-sialidase family together with other proteins such as gp85, gp82, TSA-1, and trans-sialidases. This superfamily shares common motifs with bacterial neuraminidases; however, all members of this superfamily contain a conserved sequence (VTVXNVFLYNR) that is absent in bacterial neuraminidases [32]. The members of this group are expressed on the parasite's cell surface and their concentration is stage-specific [33]. Tc85 forms a population of heterogeneous GPI-glycoproteins with similar molecular masses but different isoelectric points [34–37]. Using monoclonal antibodies that recognize Tc85 glycoproteins, like H1A10, host cell invasion was inhibited by 50%–96% [31, 34]. The Tc85 family is capable of binding to different host receptor molecules either located on the cell surface, like host cell cytokeratin 18 [14], or belonging to components of extracellular matrix, like fibronectin [30] and laminin [38], since this family is composed of multiadhesive glycoproteins.

Two groups of glycoproteins, gp82 and gp35/50, are also involved in parasite invasion. Both proteins are expressed on the surface of metacyclic trypomastigotes [39]. These glycoproteins constitute the main surface molecules of the metacyclic form in different *T*. *cruzi* strains and appear to be highly immunogenic, since mouse immunized with heat-killed metacyclic trypomastigotes produce antibodies which predominantly recognize these antigens and lyse the metacyclic forms in a complement-dependent reaction [39]. Gp82 is present only in metacyclic trypomastigotes, since antibodies against this protein did not produce any reaction towards amastigotes, epimastigotes, or tissue culture-derived trypomastigotes [40]. Ruiz and colleagues [21] demonstrated that purified gp82 binds less efficiently to HeLa cells than gp90 or gp35/50, but gp82 is capable of activating a Ca^2+^ signaling in this cell. In 1998, Favoreto and colleagues [41] demonstrated that gp82 is the signaling receptor that mediates protein tyrosine phosphorylation which is necessary for host cell invasion. Also, phospholipase C and IP3 are involved in this signaling cascade that is initiated with parasite cell surface by gp82 and leads to Ca^2+^ mobilization required to target cell invasion [10, 41]. Gp82 is a glycoprotein present, mainly, in the G strain while gp35/50 is mostly concentrated in the CL strain. Metacyclic trypomastigotes of the CL strain trigger the Ca^2+^ signaling pathway in host cells following parasite adhesion mediated by gp82 [21, 42, 43]. Gp35/50 molecules are not as effective as gp82 in promoting invasion, probably due to their poor Ca^2+^ signal-inducing activity. These mucin-like gp35/50 molecules, abundant on the surface and resistant to protease digestion, are responsible for protecting the metacyclic trypomastigotes from destruction during infection by the oral route [7]. 

Another group of molecules present on the surface of culture trypomastigotes are the trans-sialidases. Trans-sialidase, a unique enzyme of *T. cruzi*, is a surface-bound protein which is shed by the parasite into the external milieu. This trans-sialidase is a modified sialidase that, instead of releasing sialic acid, can transfer it from sialoglycoconjugates in the host to terminal *β*-galactoses in the parasite's glycoconjugates, which are unable to synthesize these molecules [44]. This enzymatic activity is different from the known eukaryotic sialyltransferases present in the Golgi complex that exclusively use CMP-sialic acid as the donor substrate. The trans-sialidase gene family comprises at least 140 members, which can be classified into three groups according to the structure and function of the protein product [45]. Trans-sialidases are expressed by trypomastigotes and are anchored by glycosylphosphatidylinositol (GPI) to the parasite plasma membrane. They have two main regions: an N-terminal catalytic region and a C-terminal extension, which repeats 12 amino acids (SAPA repeats) in tandem. By using anti-TS monoclonal antibodies, different cell types, and parasite isolates, Prioli and colleagues [46] demonstrated that the transference of sialic acid residues from the host cell to the parasite hinders *T. cruzi* infection. Subsequently, Pereira and colleagues [47] using trypomastigotes expressing trans-sialidases (TS+) and trypomastigotes that do not express trans-sialidases (TS−) demonstrated that the TS+ population was highly invasive, whereas TS− was extremely inefficient to infect nonphagocytic cells. Sialic acid is incorporated by trans-sialidases mainly into mucins present in the plasma membrane of trypomastigotes [27]. Sialylation process in *T. cruzi* was shown to confer resistance to the human complement, which is a prerequisite for infection [48]. Although TS and sialylated glycoconjugates apparently exhibit critical functions for infection, persistence, and pathogenesis of Chagas disease, the exact molecular mechanisms of their function and the receptors for these sialylated structures on the different host cells are still unknown [49]. Jacobs and colleagues [49] demonstrated that trypanosomal TS removes sialic acid from the cell surface, thereby discharging Siglecs (sialic acid-binding Ig-like lectins) present in different cells and used by different pathogens sialylated.

Inactive trans-sialidase is a sialic acid-binding lectin that costimulates host T cells through leucosialin (CD43) engagement [50]. Analyzing inactive members of the trans-sialidase family, Todeschini and colleagues [51] demonstrated that the proteins can physically interact with sialic acid-containing molecules on host cells and could play a role in host cell/*T. cruzi* interaction. 

Present in all strains of *Trypanosoma cruzi,* Gp83 is a ligand employed by the parasite to attach and enter phagocytic and nonphagocytic cells [53, 54] and it is expressed only in infective trypomastigotes [55]. Another molecule known as penetrin, a 60 kDa protein that has an affinity to extracellular matrix elements, selectively binds to heparin, heparan sulfate, and collagen and promotes fibroblast adhesion and penetration [56]. 

Some *T. cruzi* proteases have been implicated in host cell infections like cruzipain, oligopeptidase B, and Tc80. Cazzulo and colleagues [57] isolated and characterized a lysosomal cysteine proteinase, from epimastigotes of *T. cruzi,* named “cruzipain”. This enzyme is able to degrade bovine serum albumin, hemoglobin, and azocasein at pH 5.0 [58]; contains amino acid sequences presenting considerable homology with papain and cathepsin L [59]; and is a high-mannose type of glycoprotein, as shown by binding to concanavalin A Sepharose, endo-AT-acetyl-glucosaminidase H sensitivity, and determination of oligosaccharide chain composition [59, 60]. Souto-Padrón and colleagues [60] demonstrated that cysteine proteinase is located in endosomal-lysosomal (reservosome) system of epimastigotes but is also expressed on the surface of epimastigotes and amastigote-trypomastigote transitional forms. It was also showed that addition of antiproteinase antibodies to the interaction medium significantly inhibited the ingestion of parasites by macrophages. This cysteine protease is secreted through the flagellar pocket of *T. cruzi* and has been described to cleave host high molecular weight kininogen to generate short-lived kinins which bind to the bradykinin receptor to stimulate IP3-mediated Ca^2+^ release [61, 62]. 

Oligopeptidase B, an 80 KDa cytosolic serine endopeptidase, is secreted by trypomastigotes of *T. cruzi* [63, 64]. This soluble factor is generated by the action of an alkaline peptidase on precursors present only in infective trypomastigotes [64] and this peptidase is seemed to indirectly induces [Ca^2+^]_*i*_-transients during *T. cruzi* invasion [64, 65]. 

Tc80 is a prolyl oligopeptidase, a member of the serine protease family that hydrolyses human collagen types I and IV at neutral pH and also fibronectin which is important for the parasite's transit through the extracellular matrix [66, 67]. Grellier and colleagues [67] using selective inhibitors for Tc80 showed that the parasite's entry into the host cell was blocked, indicating that this molecule could be used as a possible good target for Chagas' disease chemotherapy.

In the case of amastigote, surface components that are involved in attachment and internalization in host cells have not yet been well identified. Evidences indicate that a carbohydrate epitope defined by a monoclonal antibody 1D9, abundant in lineage 1 of *T. cruzi* [68, 69], could be implicated in this process since Mab 1D9 specifically inhibits parasite invasion [68]. Recently, da Silva and colleagues [70] described a 21KDa ubiquitous protein secreted by extracellular amastigotes. Pretreatment of host cells with P21-His_6_ inhibited cell invasion by extracellular amastigotes. On the other hand, when the protein was added to host cells at the same time as amastigotes, an increase in cell invasion was observed, suggesting that p21 might be involved in *T. cruzi* cell invasion.

### 3.2. Host Cell Molecules

Since *T. cruzi*'s entry process is a multifactorial process, many molecules that are present in the membrane of the host cell, are potential partners for recognition. These factors can vary depending on the cell type involved. During initial studies, Kipnis and colleagues [71] showed that macrophages pretreated with trypsin, antimacrophage serum or with the incubation of both parasites and cytochalasin B had no effect on parasite uptake. But previous macrophage treatment with cytochalasin B blocked parasite invasion. On the other hand, Henriquez et al. [72] showed that host cells (like Vero and chick muscle cells) pre-treated with concanavalin A, phytohemaglutinin, wheat germ agglutinin, or ricin impaired trypomastigote invasion. The authors also showed that trypsin and periodate treatment also inhibited parasite infection, showing the participation of proteins and glycoproteins during the process. 

One class of receptors present in mammalian cells is represented by lectin-like molecules. Lectins are sugar-binding proteins which are highly specific for their sugar moieties and are involved in attachment between pathogens and host cells [73]. Galectin-3 [74], a *β*-galactosyl-binding lectin, is a type of lectin involved in *T. cruzi* attachment has been suggested to mediate parasite attachment and entry in dendritic cells [75] and in smooth muscle cells [76]. Carbohydrate residues present in the plasma membrane of mammalian cells can function as receptors. Using electron spectroscopic imaging (ESI) and lectins like WGA, RCA I, and ConA, Barbosa and de Meirelles [77] detected galactosyl, mannosyl and sialyl residues in regions of host-cell plasma membrane that are internalized together with the parasite. Glycosylation mutants of Chinese hamster cells (CHO) showed that adhesion and invasion of *T. cruzi *was impaired in cells that express very few sialic acid residues. If the deficient cell line was incubated in the presence of exogenous fetuin and *T. cruzi *trans-sialidase, the infection process was similar to that observed in parental cells [78].

Integrins, receptors that mediate attachment between two cells or cell and extracellular matrix, are involved in the invasion processes. Tc85, that is present at *T. cruzi *membrane and has been associated with parasite invasion, contains fibronectin-like binding sequences [79] and a laminin-binding domain [78]. Besides functioning as a cellular link to laminin or fibronectin, integrins function as receptors that can activate PI-3 kinase signaling pathways. Tc85 could bind to cytokeratin 18, a cytoskeletal protein that was suggested to function as a *T. cruzi* receptor [42]. However, when cytokeratin 18 expression was silenced by RNAi, trypomastigote binding to host cells was not affected, although intracellular growth of amastigotes was impaired [80]. 

Another molecule present on the host cell surface and involved in trypomastigotes' entry is the TGF receptor. Signal transduction through TGF-*β* receptors facilitates *T. cruzi *entry into epithelial cells [15, 81]. However, the molecule present in trypomastigotes that is capable of binding to the TGF *β* receptor is not yet known. Scharfstein and Morrot [15] proposed that infective stages of *T. cruzi *secrete a TGF*β*-like molecule or a factor capable of activating latent host TGF*β*. The exposure of phosphatidylserine on the surface of *Trypanosoma cruzi *trypomastigotes [82] and its deactivating effect on macrophages by the induction of TGF *β* suggests that phosphatidylserine is a possible activator of TGF *β* [82].

The bradykinin receptors are another class of receptors used by trypomastigotes to penetrate mammalian cells. They are coupled to the heterotrimeric protein G and are formed by two subtypes: the bradykinin 2 receptor, which is constitutively expressed by cardiovascular cells, and the bradykinin 1 receptor whose expression is upregulated in injured tissues [62]. The use of chinese hamster ovary (CHO) cells overexpressing bradikinin2 receptor showed that trypomastigotes' invasion is modulated by combinated activities of kininogens, kininogenases, and kinin degrading peptidases. Trough bradykinin 2 receptor, trypomastigotes elicit vigorous intracellular free calcium transients.

 Present in neuronal and dendritic cells, the nerve growth factor receptor TrkA leads to *T. cruzi *invasion through trypomastigote trans-sialidase binding [83]. Moreover, many types of neurons and glial cells express a neurotrophic receptor called TrkC (tyrosine kinase C). *T. cruzi* binds to TrkC to maximize host-parasite equilibrium in the nervous system and this interaction is mediated by trans-sialidase/parasite-derived neurotrophic factor (PDNF), previously identified as a TrkA ligand. Thus, TrkC is a new neurotrophic receptor that *T. cruzi* engages to promote the survival of neuronal and glial cells [83].

In 2001, Campos and colleagues [84] described that *Trypanosoma cruzi* derived glycosylphpsphatidylinositol (GPI) linked to mucin-like glycoproteins and glycoinositolphosphoslipids (GIPLs) were recognized by TLR2/CD14 of host cells. Toll-like receptor activates nuclear factor *κ*B and interferon regulatory factor dependent pathway [85]. *T. cruzi*-derived GPI anchors can also phosphorylates mitogen-activated protein kinases (MAPKs) and I*κ*B in macrophages [86]. Maganto-Garcia and colleagues [87] demonstrate that Toll-like receptors 2 induces phagocytosis of trypomastigotes by stimulating the activation of Rab5. Tissue-culture derived trypomastigotes initiate an inflamation process after triggering Toll-like receptor-2 in macrophages [88]. 

The other Toll-like receptors which recognize *T. cruzi* are Toll-like receptors 4 and Toll-like receptors 9. The GIPL ceramide isolated from epimastigotes of *T. cruzi* is suggested to interact with Toll-like receptor 4. Toll-like receptor 9 is known to be activated by methylated CpG-rich DNA and *T. cruzi* DNA in macrophages [88].

## 4. Morphology of the Adhesion and Internalization Process

Since *Trypanosoma cruzi* infects several cell types and uses different mechanisms to invade host cells, a wide type of morphological events could be observed. Our group demonstrated by field emission scanning electron microscopy that even after a short interaction time, all developmental stages of *T. cruzi *are readily ingested by the macrophages. After 15 minutes of interaction, a variety of interaction types could be distinguished morphologically. In the case of trypomastigote forms, most of them entered the macrophages with their posterior region (65%). While amastigotes stages did not show a preferential region of entrance epimastigotes, the noninfective stage of *T. cruzi*, were internalized mainly via the flagellar region. The macrophages' plasma membrane are covers the parasite by forming a funnel-like structure or a structure described as a coiled-coil phagosome [29]. The use of drugs which inhibits PI 3 Kinase (PI3K) activity demonstrated that trypomastigotes entered mainly through the posterior region, in drug-treated cells the trypomastigotes entered mainly through the anterior region. However, in the case of epimastigotes, PI3K inhibitors did not interfere with the entry pattern. Inhibition of PI3K inhibited the complete sealing of surface projections that participate in the endocytic process.These results suggest that when PI3K is blocked, trypomastigotes use another type of ligand, exposed on their anterior region to initiate the invasion process [29].

## 5. Triggering of the Endocytic Activity

**Figure 3:**
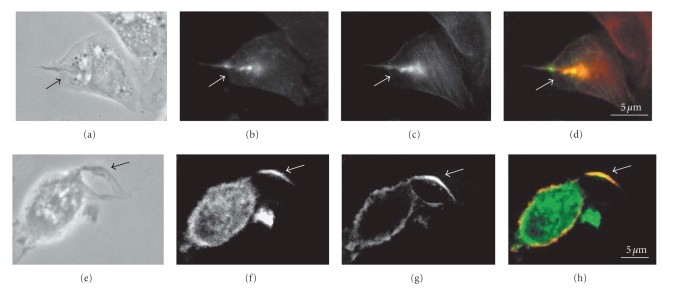
Immunofluorescence microscopy colocalization (arrows) of phosphorylated proteins (b, g), detected using antiphosphotyrosine antibody, actin filaments (c), detected using phalloidin, and PI3 kinase (f) detected using anti-PI3 Kinase antibody during penetration of *T. cruzi* into macrophages. The overlay images (d, h) show the areas of colocalization demonstrating that phosphorilated proteins and microfilaments participate in internalization of *T. cruzi* trypomastigotes by macrophages. Bars = 5 *μ*m (after [9]).

Following binding and recognition of the parasite by the host cell surface, a series of cell signaling processes take place which culminate in the invasion of the host cell. The available evidence indicates that the trypomastigote stage of *T. cruzi* uses several mechanisms to signal and invade host cells. One entry mechanism is phagocytosis/macropinocytosis, where the cells emit pseudopods and there is participation of actin filaments. In professional phagocytes (such as macrophages) the activation of tyrosine kinase proteins was observed, followed by the recruitment of PI-3 kinase and actin filaments (this process has been associated with the mechanism of phagocytosis) at the spot of entry of the trypomastigote [9], showing that phagocytosis is the main mechanism of *T. cruzi *entry in macrophages (Figure 3). In another mechanism known as endocytosis, emission of pseudopods does not take place, but present participation of actin filaments. Trypomastigotes can also entry by invagination of the membrane, without participation of actin filaments. This latter process has been regarded as an active mechanism for the entry of the parasite with waste of energy [89].

In the initial moments of the recognition between *T. cruzi* and the host cell, a transient increase of cytoplasmic levels of calcium (in both parasite and host cell) occurs [10, 90, 91]. If this transient increase in cytoplasm calcium is blocked by treatment with thapsigargin, for example, a reduction of parasite invasion was observed [92]. Also, in nonprofessional phagocytic cells there is a recruitment of lysosomes to the place of invasion of the parasite, although this phenomenon represents about 20% of entering parasites (it occurs in about 20%). The lysosome-dependent pathway is initiated by targeted Ca^2+^-regulated exocytosis of lysosomes at the plasma membrane. Another pathway used for parasite internalization in nonphagocytic cells is the lysosome-independent pathway. In this model, parasites enter cells through plasma membrane invaginations that accumulate PIP3, the major product of class I PI3K activation. As a result of this mode of entry, around 50% of total internalized parasites are contained in vacuoles enriched in plasma membrane markers and about 20% are in early endosomes (EEA1 labeled) at 10 min postinfection. In this case, the immature vacuole is enriched with lysosomes within 60 min [93].

 Transient increases of calcium in the cytoplasm of the host cell, after the interaction with trypomastigotes of *T. cruzi*, has been shown to cause a reorganization of the actin cytoskeleton [92, 94]. Also, depolymerization of actin filaments at the parasite site of entry may enhance parasite invasion [95]. Several studies using host cells treated with cytochalasins D or B (agents that depolymerize actin filaments) before the process of interaction with trypomastigote forms showed controversial data: some authors say that the treatment inhibits the entry of trypomastigotes [11, 96, 97], others describe a sharp increase in entry [98], while others [72] showed almost no effect. However, when analyzing all the data, we see that the time of treatment, interaction times with trypomastigotes, the nature of host cells and the strains of *T. cruzi* varied among the experiments. In addition, we can not exclude the possibility that attached parasites were considered as ingested.

The entry of the trypomastigotes activates signaling processes in the host cell, leading to the invasion of the parasites. In professional phagocytes the activation of tyrosine kinases, recruitment of PI-3 kinase and actin at the parasite entry site occur [29, 99] (Figure 3). 

Activation of tyrosine kinases does not occur in non-professional-phagocytic cells, as shown in studies where inhibitors of these enzymes were used resulting in no reduction of the invasion process. However, the activation of PI-3 kinase occurs [28] and this activation seems to be a regulator of the phagocytosis with the participation of the host cell lysosomes [28], including the involvement of tyrosine phosphatase in this process. 

**Figure 4:**
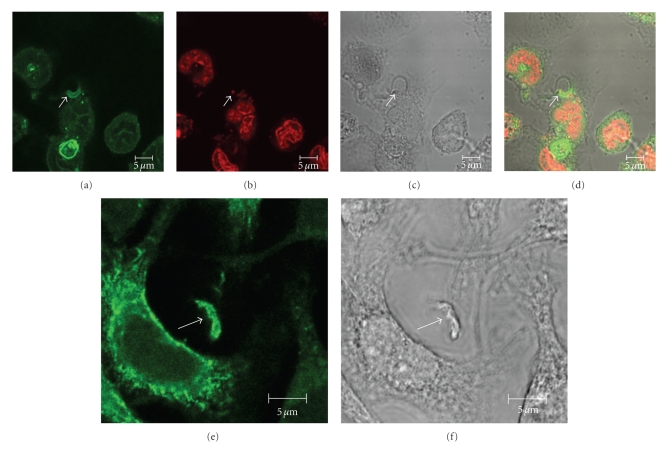
Imunofluorescence microscopy localization of GM1 (a–d) and flotillin 1 (e-f) during internalization of *T. cruzi* trypomastigotes by macrophages suggests the participation of membrane microdomins in this process. (a–d) colocalization of GM1, using cholera toxin subunit B (a) and an intracellular parasite ((c): arrow). (b) shows labeling of the nucleus and kinetoplast labeled with propidium iodide. (c) corresponds to a DIC image; (d) is a merge image. (e-f): Colocalization of flotillin1 (a), detected using a specific antibody, and trypomastigotes ((b): arrows) bars – 5 *μ*m (after [16]).

Host cell plasma membrane microdomains were also shown to be involved in *T. cruzi* entry both in nonphagocytic and phagocytic cells [16, 100]. Both groups demonstrated that cholesterol, the major components of membrane rafts and molecular markers of this domain like flotillin1 colocalized with trypomastigote and amastigote entry sites, suggesting the participation of microdomains in *Trypanosoma cruzi* invasion (Figure 4).

## 6. Parasitophorous Vacuole Assembly

**Figure 5:**
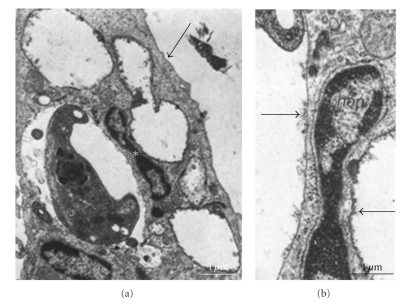
Macrophages first labeled with ferritin-ConA at 4°C, incubated with *T. cruzi *at 4°C, and then incubated for 30 min at 37°C, after which the cultures were fixed. The ferritin particles (arrows in (a) and (b)) associated with the membrane of vacuoles without parasites are uniformly distributed, showing a patchy distribution in those vacuoles that contain parasites. (b) High magnification of the area marked with an asterisk in (a), showing in detail the membranes of two different vacuoles. *hcn, *host cell nucleus; *p, *parasite; and *pvc, *parasite-containing vacuole. Bars = 1 *μ*m (after [17]).

After the recognition process between *T. cruzi *trypomastigote and a host cell, the parasite may or may not invade it. It was shown that sugar residues are important during recognition and invasion. So, with the use of labeled Concanavalin A (fluorescein or ferritin labeled) it was possible to show that Con A binding sites on the macrophage surface were internalized together with the trypomastigote, being observed associated with the parasitophorous vacuole membrane [17] (Figure 5). Trypomastigote interaction with cationized ferritin prelabeled macrophages (at 4°C) showed that the parasitophorous vacuole containing the parasite was negative for cationized ferritin, although this marker can be observed inside the cytoplasmic vesicles. Macrophages incubated with cationized ferritin and horseradish peroxidase at 37°C, ingested both makers and concentrated them in endocytic vacuoles. When these pre-labeled macrophages were allowed to interact with trypomastigote stages, the parasites were observed only in vacuoles labeled with horseradish peroxidase but not with cationized ferritin [101]. This observation suggests that the parasite may modulates the parasitophorous vacuole membrane composition.

**Figure 6:**
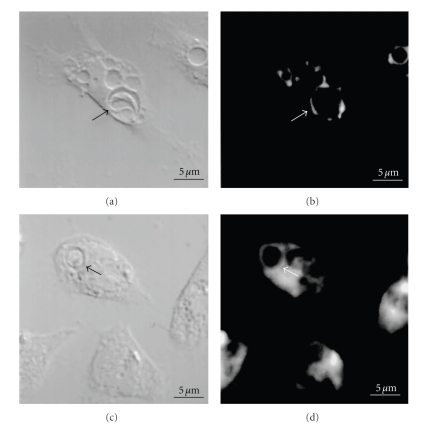
(a, b) DTAF (sialic acid marker) labeled macrophages were incubated with trypomastigote forms of *Trypanosoma cruzi *for 1 hr at 37°C. Observation by confocal laser scanning microscope (CLSM): DIC image (a) and corresponding fluorescence (b) images. Observe the labeling of the membrane lining the parasitophorous vacuole containing a trypomastigote form (arrow); (c, d) macrophages labeled with PKH-26 (lipid marker) were allowed to interact with trypomastigote forms of *T. cruzi *for 1 hr at 37°C. Observation by CLSM: DIC (c) and corresponding fluorescence images. Observe labeling of the plasma membrane in the parasitophorous membrane containing a trypomastigote form (arrow). Bar: 5 *μ*m (after [18]).

**Figure 7:**
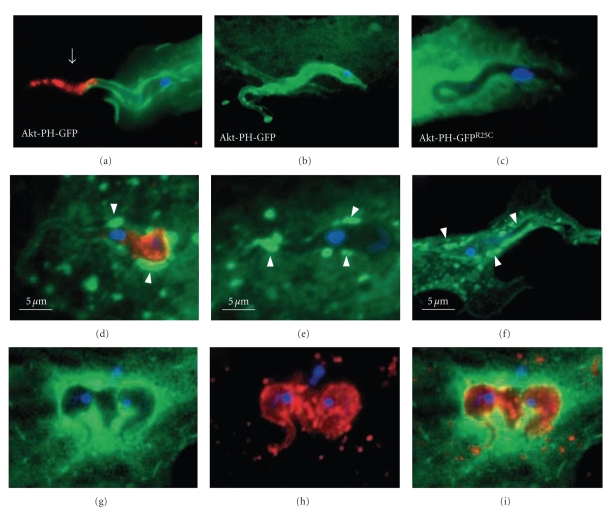
Trypomastigote vacuole is enriched in AKT-PH-GFP that binds to posphatidylinositolbiphosphate (PtdIns(3,4)P_2_) and phosphatidylinositoltriphosphate (PtdIns(3,4,5)P_3_) that are products of PI3 Kinase (a–c) and also labeled to EEA1 and Rab 5 (d–f) and Lamp-1 (g–i). (a–c) rapid accumulation of PtdIns(3,4,5)*P*3/PtdIns (3,4)*P*2 at the *T. cruzi *invasion site. Fluorescence images of (a) CHO cells or (b) primary rat cardiomyocytes transiently expressing Akt-PH-GFP following incubation with infective *T. cruzi *trypomastigotes for 15 minutes. (c) *T. cruzi *invasion of CHO cells expressing Akt-PH-GFP^R25C^ which fails to bind to PtdIns (3,4,5)*P*3/PtdIns(3,4)*P*2. (d)–(f): early association of *T. cruzi *with early endosomes is minimal and precedes lamp-1 acquisition. Immunofluorescence staining of extracellular *T. cruzi *(arrowheads) following infection of CHO cells transiently expressing Rab5-GFP (green). Host cell and parasite DNA is visualized with DAPI (blue). (g–i) lamp-1 association with the Akt-PH-GFP-enriched *T. cruzi *vacuole occurs after parasite entry. L6E9 myoblasts expressing Akt-PH-GFP (green) were infected with *T. cruzi *for 60 minutes and stained with anti-lamp-1 (red) and DAPI (blue). Bars = 5 *μ*m (after [28]).

**Figure 8:**
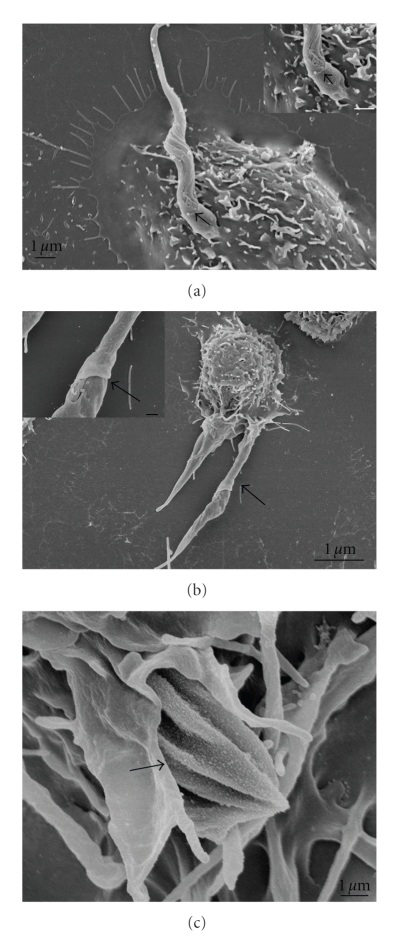
Field emission scanning electron microscopy of the interaction between peritoneal macrophages treated with dynasore 60 *μ*M (for 20 minutes) and allowed to interact with *T. cruzi *trypomastigotes (a), epimastigotes (b), and amastigotes (c). All parasite evolutive forms were partially recovered by the macrophage plasma membrane indicating that the blockage of GTPasic dynamin activity did not impair the pseudopod extension, impairing only the complete vacuole formation. The interaction time was enough to complete the parasite entry into control macrophages. Bars = 1 *μ*m (after [29]).

In 1987, De Carvalho and De Souza [102] showed that opsonized trypomastigote and epimastigote stages interacting with macrophages activate NAD(P)H oxidase at the host cell membrane and that this enzyme is kept activated inside the parasitophorous vacuole (PV). The first study describing the PV membrane composition used a macrophage cell line and showed: (i) the presence of Fc receptors [103] if trypomastigotes were opsonized with anti *T. cruzi *antibodies; (ii) *β* 1 integrin and lysosomal membrane glycoproteins (lgp); and (iii) the presence of complement receptors (CR3), Fc receptors if epimastigotes were opsonized with anti-*T. cruzi* antibodies; *β* 1 integrin and lysosomal membrane glycoproteins (lgp). Using muscle cells, Barbosa and de Meirelles [77] showed with Thiéry staining that glycoconjugates were present in the parasitophorous vacuole membrane. They also used ferritin labeled RCAI to detect galactosyl residues and showed that in muscle cells these residues accumulated in the parasite adhesion region and these residues were internalized during parasite invasion [77]. In 1994, Tardieux et al. [98] and Rodríguez et al. [104] showed that lysosomes migrate early to the parasite entry site in nonphagocytic host cells, contributing with membrane during the PV formation. They pointed out, as requirements for parasite entry, the participation of microtubules and kinesin in the lysosomes migration from the perinuclear region to the cell periphery [105]. The authors also show that lysosome-membrane fusion is dependent on calcium [105]. Ochatt et al. [106] showed, using macrophages that GTP-regulated factors, but not calcium-regulated elements, were involved in an early inhibition of phagosome-lysosome fusion in *T. cruzi *infected macrophages. Carvalho et al. [18] using fluorescent markers showed that host cell membrane lipids, proteins and sialoglycoconjugates contribute to the membrane lining the PV, which contains epimastigotes and trypomastigotes ingested by macrophages (Figure 6). Lysosome fusion at the parasite entry site during early infection of macrophages by trypomastigotes has not been clearly shown. Using nonphagocytic GFP-Rab5 transfected cells and confocal microscopy, Wilkowsky et al. [107] demonstrated the presence of Rab5 in early PV containing *T cruzi*, indicating that some PV fuse first with endosome vacuoles. Woolsey et al. [28], using shortly infected nonprofessional phagocytic cells, showed that 50% or more of invading *T. cruzi *trypomastigotes use host cell plasma membrane during the PV formation. They suggested that this process was facilitated by the depolymerization of host cell actin microfilaments. They also showed that this vacuole is enriched in products from PI 3-kinase and negative for lysosomal markers; approximately 20% of *T. cruzi* containing vacuoles were positive to EEA1 and Rab 5 and approximately 20% of *T cruzi* containing vacuoles were positive for Lamp-1 (Figure 7). Questioning *T cruzi *early residence in a phagolysosome, Andrade and Andrews [108], blocked *T. cruzi *lysosome mediated invasion and showed that the parasites were not retained inside the host cell. They concluded that the phagolysosome fusion is essential for parasite retention inside host cells and development. Concerning the lysosome fusion at the parasite entry site, Tyler et al. [109] showed that the parasitophorous vacuole containing *T. cruzi* acts as a secondary microtubule organizing center. More recently, Romano et al. [110] showed that the PV containing *T. cruzi* is decorated by the autophagic protein LC3. This paper also pointed out some interesting observations: (i) host cell starvation or pharmacological induction of autophagy before the infection with *T. cruzi* significantly enhances the number of infected cells, while inhibitors of this process inhibited parasite invasion; (ii) the absence or reduction of two proteins required in the initial step of autophagosome formation (Atg5 and Beclin 1) reduces both the parasite entry and Lamp-1 association with the PV; and (iii) autolysosomes are recruited to the parasite site of entry. Fernandes et al. [100] and Barrias et al. [16] observed in the PV containing *T. cruzi* the presence of GM1, flotillin and caveolin 1 shortly after infection, thus suggesting the presence of microdomains in the membrane lining the *T. cruzi* PV. Dynamin is essential to vacuole parasitophorous formation since the blockage of its GTPasic activity by dynasore (a dynamin inhibitor) impairs the parasite internalization [29] (Figure 8).

## 7. Lysis of the Parasitophorous Vacuole (PV) Membrane

**Figure 9:**
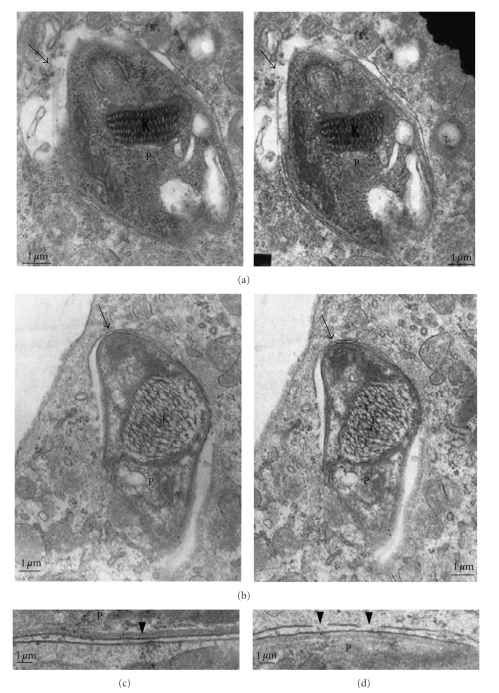
Transmission electron microscopy (TEM) of thin sections of macrophages infected with trypomastigote forms of *T. cruzi*. Micrographs taken at different inclination angles of the section. Focal disruption of the membrane lining the vacuole is observed (arrows in (a) and (b)) and especially in (c) and (d); K = kinetoplast, P = parasite. Bars = 1 *μ*m (after [52]).

Trypomastigote stages of *T. cruzi* use different receptor0073/linkers to get into host cells. Regardless of the mechanism used (either by fusion of lysosomes at the site of entry, by participation of components of the plasma membrane or by initial fusion with endosome compartments at the site of invasion), the parasite will be located in a vacuole. Studies show that some molecules are excluded when the vacuole is being formed. Some authors refer to the process of lysis of the PV membrane as an “escape” of the parasite from the parasitophorous vacuole. We prefer to characterize this process as a consequence of disintegration of the parasitophorous vacuole membrane. More studies are necessary to better characterize the mechanism involved in this important step of the *T. cruzi* life cycle. In the PV, trypomastigote forms release trans-sialidase/neuraminidase, that will remove sialic acid residues from the PV membrane making it sensitive to the action of Tc-Tox (a peptide that has homology with the factor 9 of the human complement) [111]. At the acidic pH of PV, this molecule will begin to destroy, maybe by pore formation, the PV membrane [8, 112, 113] (Figure 9). We suppose that the formation of these small pores, associated with the action of secreted enzymes, like transialidase/neuraminidase, by the parasite, will lead to the fragmentation of the PV membrane. Host cell treatment with drugs that raise the intracellular pH delayed the fragmentation of the PV membrane [114]. On the other hand, observations using CHO cells that are deficient in sialylation showed that the absence of sialic acid makes the PV membrane more sensitive to lysis [115]. The presence of sialic acid residues seems to protect the lysosome membrane from lysis. 

The amastigote stage was also shown to be infective to phagocytic [52, 116] and nonphagocytic cells, an important process during the cycle in different hosts [117]. Observing blood smears of mice infected with *T. cruzi*, we could detect the presence of an amastigote (or amastigote-like form) in these infected mice. Stecconi-Silva et al. [118] showed that amastigote was recognized by monoclonal antibody against C9 complement protein, suggesting that TcTox is also present and active in intracellular amastigotes. Amastigotes may be able to use transialidase and TcTox to lysis the parasitophorous vacuole membrane in a process faster than occurs with trypomastigote forms, although Stecconi-Silva et al. [118] showed negligible transialidase and hemolytic activity (TcTox activity) by amastigote forms only 12 hs after incubation with red blood cells. They also showed that raising the host cell cytoplasm pH using chloroquine, the time metacyclic remains in the parasitophorous vacuole increased two times, with no effect on amastigote. The kinetic of metacyclic trypomastigote and amastigote stages escaping from vacuole after raising host cell cytoplasm were not affected.

**Figure 10:**
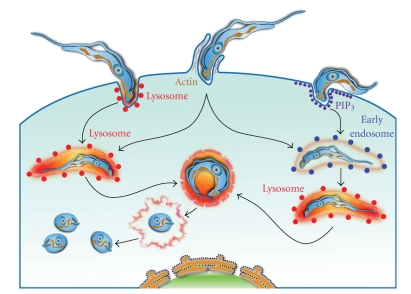
Model of *T. cruzi* invasion. The model indicates three distinct mechanisms of *T. cruzi* entry into host cell. (a) the lysosome-dependent pathway is initiated by targeted Ca^2+^-regulated exocytosis of lysosomes in the plasma membrane; (b) in the actin dependent pathway trypomastigotes penetrate into a host cell through a plasma membrane expansion that culminates in assembly of a parasitophorous vacuole. Either learly endosomes or lysosomes can fuse with the parasitophorous vacuole; (c) in the lysosome-independent pathway, parasites enter cells through plasma membrane invaginations that accumulate PIP_3_ (product of class I PI3K activation). Subsequently, internalized parasites are contained in vacuoles formed from the plasma membrane and it maturates with the acquisition of early endosome markers (rab5 and EEA1) and subsequently with the acquisition of lysosome markers. Later on, the the trypomastigote form gradually transform into a amastigote form with simultaneous lysis of the parasitophorous vacuole membrane. Then, amastigotes in direct contact with the cytoplasm start to divide.

The trypomastigotes which started a process of differentiation into amastigotes, while they are still located inside the PV during its membrane fragmentation was observed by transmission electron microscopy around 2 hours after infection [8]. In Figure 10, we have a scheme that sinthesized the mechanisms used by trypomastigotes to start an infection in a host cell.

## 8. Perspectives

Since the discovery of *Trypanosoma cruzi* and Chagas' disease a century ago, many groups have concentrated their efforts on understanding the parasite's cell biology and its interaction with different host cells, including the vector cells. Although significant progress has been achieved on the identification of surface parasite molecules involved in the interaction process very little information is available on the nature of the host cell ligands involved in such processes. This is certainly an important area for future research. The available information also points in the direction of the co-existence of several processes of penetration of *T*. *cruzi* into different cells. It is not yet clear what leads the parasite to select among these mechanisms. We also have to better understand the biogenesis of the parasitophorous vacuole. Is there a signaling dictated by the parasite that leads to the parasitophorous vacuole disintegration? Are there molecules from the host cell cytoplasm that cooperate with this process? Can this process be inhibited, and what would the consequence to the parasite inside this vacuole be? Besides this, little is known about parasite differentiation in the mammalian and in the insect vector. Is nutrient privation the only mechanism responsible for the differentiation process? What triggers the rupture of the host cell and the release of infective stages into the intercellular space? Several of these questions will be the subject of further investigation in the next few years.
